# Physical Treatment in Heart Disease

**Published:** 1896-02-22

**Authors:** 


### PHYSICAL TREATMENT IN HEART DISEASE.

Dr. Percy Wilde writes to say that the criticism of his paper on physical treatment in heart disease,[1] which has been made by Miss Theodora Johnson, to the effect that he associated with a Swedish exercise methods completely at variance with the fundamental principles upon which Ling's treatment, must have been founded on a misconception of his meaning, for the very object of his article was to call attention to the error of that "fundamental principle," and he goes on to defend his proposition that during the exercises which he advises the breath should be held. We cannot now enter into this matter further than to say that while many will agree with him that, so far as the performance of varied exercises for the development of the body is concerned, it is well to hold the breath during effort, many will also assert that, when exercises are performed for the benefit of the heart rather than the muscles, easy breathing should be continued, and that effort should never be carried to such an extent as to require holding of the breath.

[1] The Hospital, February 1st, 1896.

